# Formaldehyde Vapor Concentration in Electronic Cigarettes and Health Complaints of Electronic Cigarettes Smokers in Indonesia

**DOI:** 10.1155/2018/9013430

**Published:** 2018-07-11

**Authors:** Kusuma S. Lestari, Mika Vernicia Humairo, Ukik Agustina

**Affiliations:** ^1^Department of Environmental Health, Faculty of Public Health, Universitas Airlangga, Surabaya 60115, Indonesia; ^2^Faculty of Public Health, Universitas Airlangga, Surabaya 60115, Indonesia

## Abstract

Electronic cigarettes regulation in Indonesia has not been set yet. In the last 4 years the electronic cigarettes have been widely distributed and used in Indonesia. Electronic cigarettes contain nicotine, propylene glycol, glycerol, liquid flavors, etc. All ingredients produce vapor when heated. Vapor and particles from electronic cigarettes affect the human health. Formaldehyde is known as a product of propylene glycol and glycerol vapor degradation. Formaldehyde is one of the chemical agents categorized as carcinogen. The aim of the research was to analyze the identification of formaldehyde vapor concentration and health complaint of electronic cigarettes smoker. The research was conducted in Surabaya city, Indonesia, from October 2015 to December 2016. The research used cross-sectional approach. Sample was obtained by purposive sampling that fulfilled samples inclusion criteria. The variables were the onset of smoking electronic cigarettes, smoking frequency of electronic cigarettes, formaldehyde vapor concentration, cotinine urine, and health complaint of electronic cigarettes smoker. The result showed that formaldehyde concentration in six vapors varied while cotinine urine mostly was positive. It is suggested to educate people about hazard of electronic cigarettes and to conduct further research to identify chemical agent in electronic cigarettes.

## 1. Background/Objectives and Goals

Electronic cigarette was developed in the beginning in 2004 as an alternative of nicotine replacement therapy in liquid to reduce tobacco consumption [1]. Electronic Nicotine Delivery System (ENDS) is a device to convert chemical agent to vapor and divert it from lungs. The device consists of rechargeable batteries, electronic regulator, and a container of liquid that will be evaporated [2].

Electronic cigarettes are produced by industries in order to stop smoking or as an alternative of tobacco cigarettes because it does not produce smoke. It is added more than 8,000 flavorings and one of them is nicotine. Nicotine is addictive to a person who wants to stop smoking, especially young people. According to South-East Asia Region (SEARO) (2013) the total of electronic cigarette smokers has doubled in adults from 2008 to 2012 [3]. Centers for Disease Control and Prevention (CDC) researchers, Food and Drug Administration (FDA), and Georgia State University stated that the number of teens who had never smoked but use electronic cigarette increased three times during the year 2011-2013. The number of teens who do not smoke conventional cigarettes but use electronic cigarettes increased from 79,000 in the year 2011 and became 263,000 in 2013 [4]. In 2013 the use of electronic cigarettes was doubled among US middle and high school students during 2011–2012. Moreover, in 2012, about 160,000 students who had not been using conventional cigarettes were using electronic cigarettes [5]. Intention to smoke conventional cigarettes was 43.9% among electronic cigarettes smoker and 21.5% among non-electronic cigarettes smoker [4]. In UK, in 2013, about 1.3 million smokers used electronic cigarettes while in previous year there were about 700,000. Several studies in US showed that about 38% smokers have used electronic cigarettes in order to quit smoking [5].

Electronic cigarette was first introduced in China in 2003 and was distributed online afterwards [2]. Electronic cigarette distribution extends to several countries and Indonesia is one of them. In some countries the regulations related to the distribution of electronic cigarettes are different: they are considered as illegal goods in some, are allowed (no regulation), and are allowed under certain conditions and have been organized by the Ministry of Health as a registered medicine (not freely traded) in others. In Indonesia, the regulations related to the distribution and use of electronic cigarette have not been set yet. Indonesia Food and Drug Agency stated that electronic cigarettes that have been distributed in some cities are illegal and unsafe products. This product has not been tested clinically; therefore it is dangerous. World Health Organization (2014) stated that products containing nicotine in various forms such as electronic cigarette are unsafe to use which can cause addiction to nicotine so World Health Organization (WHO) recommends banning the distribution [1].

Electronic cigarette contains nicotine, propylene glycol, and some other substances. During vaping the gas phase 1.2-propanediol, glycerin, and nicotine were found. The concentration of Polycyclic Aromatic Hydrocarbons (PAH) in indoor air increased by 20% to 147 ng/m^3^ and aluminum showed a 2.4-fold increase; particle number concentration (PNC) ranged from 48,620 to 88,386 particles/cm^3^ [6]. Pollutant is containing Volatile Organic Compounds (VOCs), carbonyls, PAH, nicotine, Tobacco-specific nitrosamine (TSNAs), and glycols [7]. At least 10 chemicals identified in mainstream (MS) and secondhand (SS) Electronic Smoking Device (ESD) aerosols are on California's Proposition including acetaldehyde (MS), benzene (SS), cadmium (MS), formaldehyde (MS, SS), isoprene (SS), lead (MS), nickel (MS), nicotine (MS, SS), N-nitrosonornicotine (MS, SS), and toluene (MS, SS) [8].

Health risks from electronic cigarette smoking are derived from the ability of vapors that contain fine particles of the nicotine and other harmful substances to enter into lungs. Nicotine increases the adrenaline, increasing blood pressure, and pulse. Propylene glycol at high levels causes irritation if inhaled [2]. Vapor of electronic cigarette contains some toxic substances and levels of toxic substances 9-450 times lower than cigarette smoke [9]. Another substance in electronic cigarette is formaldehyde which is categorized as a carcinogen. Formaldehyde is known as a result of the degradation products of propylene glycol and glycerol during the process of evaporation and incomplete combustion. In many samples of the particulate matter (i.e., the aerosol) in electronic cigarettes, more than 2% of the total solvent molecules have converted to formaldehyde-releasing agents, reaching concentrations higher than concentrations of nicotine. This occurs because of propylene glycol and glycerol is heated using oxygen [10]. When propylene glycol is heated it may change the chemical composition and produce propylene oxide in a little amount which is known as carcinogen [11]. In the past four years, the electronic cigarette's community in Surabaya city has been developed. The aim of research was to analyze the identification of formaldehyde vapor concentration and health complaint of electronic cigarettes smoker.

## 2. Materials and Methods

The research used cross-sectional approach. The research was conducted in Surabaya city, Indonesia, from October 2015 to December 2016. The sample consisted of active electronic cigarettes smokers who have been smoking for at least 2 months as members of electronic cigarette community. The inclusion criteria were being above 18 years. The sample consisted of 20 respondents and it was obtained by purposive sampling that fulfilled samples inclusion criteria.

The dependent variables were health complaints of active electronic cigarettes smoker. The independent variables were the onset of smoking electronic cigarettes, smoking frequency of electronic cigarettes, reasoning of using electronic cigarettes, being a person who asked to use electronic cigarettes, opinion on policy of electronic cigarette in Indonesia, cotinine urine, and formaldehyde vapor concentration. Data was obtained by interviews, questionnaire, and measurement.

Health complaints were obtained by interviews using questionnaire. They were subjective feelings during smoking electronic cigarettes such as irritation in nose, eye, and throat. Nose irritation included itchy nose, uncomfortable smell, and sneezing. Eye irritation included watery eye, sore eye, and reddish eye. Upper airway irritation included sore throat, dry throat, cough, and asphyxia. The onset of smoking, smoking frequency of electronic cigarettes, reasoning of using electronic cigarettes, being a person who asked to use electronic cigarettes, and opinion on policy of electronic cigarette in Indonesia were obtained by interviews using questionnaire. The onset of smoking identifies the age when the person started smoking electronic cigarettes. The smoking frequency of electronic cigarettes identifies the frequency of smoking electronic cigarettes in a day.

Formaldehyde was measured in media which did not contain formaldehyde material and was made from glass. Glass container was filled by electronic cigarette vapor by smoker. There was three smokers and each smoker was using two electronic cigarettes brands. In the first minute the smoker inhaled and exhaled into glass container. In the second and the third minutes the smoker took a break. They continued to the fourth minute to inhale and exhale and in the fifth and the sixth minutes they took a break again. This was repeated until the 60th minute. The formaldehyde device was using Airchek Sampler put inside glass container before the measurement was done. The measurement of electronic cigarettes brand was done to identify the formaldehyde concentration vapor in six brands of electronic cigarettes that are liquid local 90(VG)/10(PG), liquid USA 60(PG)/40(VG), liquid Malay 60(PG)/40(VG), liquid local 60(PG)/40(VG), liquid Malay 30(PG)/70(VG), and liquid USA 70(PG)/30(VG). Before measuring six vapors, in the beginning formaldehyde concentration was measured in empty glass container as a control. Formaldehyde vapor concentration was analyzed at Occupational Safety and Health Laboratory. The method used colorimeter, the principle of which is formaldehyde reacting with chromotropic acid-sulfuric solution to form a purple monocationic chromogen and being read by spectrophotometer at 580 nm. Sensitivity is from 0.1 *µ*g/mL to 2.0 *µ*g/mL of formaldehyde which can be measured in the color developed solution. The procedure of formaldehyde concentration measurement with colorimetric method was sampling formaldehyde from vapor with ambient air at a rate of 1 L/min for 24 hours, adding 4-mL aliquot from each of the sampling solutions into glass stoppered test tubes, adding 0.1 mL of 1% chromotropic acid reagent to the solution and mix, adding slowly and cautiously 6 mL of concentrated sulfuric acid, and reading at 580 nm in a suitable spectrophotometer using a 1-cm cell.

Cotinine urine was measured by COT rapid test cassette (urine) (200 ng/ml). The subject should sign and date the consent form. The interviewer team also has to sign and date the consent form. This research was approved by Faculty of Public Health Universitas Airlangga's committee ethical board.

## 3. Result and Discussion

### 3.1. Characteristics of Electric Cigarette Smoker

Characteristics of electronic cigarette smoker identified age and sex (Table 1). Most of them were 19 years old (20.0%).  Table 2 showed that 80.0% finished high school and 44.0% were college students.

Table 3 described that mostly they started using electronic cigarettes in 2015 (36.0%), while frequency of using electronic cigarettes (Table 4) was twice a day and three times a day (12.0%). Some respondents used electronic cigarettes unregularly, only 3 times a week, when there was available liquid, if there was a friend, or when they were stressed.

Person who asked respondents to use electronic cigarettes mostly were their friends and some respondents' initiative came from their own will.

Mostly, reasoning of smoking electronic cigarettes (Table 5) was to quit smoking conventional cigarettes (36.0%) and to try (24.0%).

### 3.2. Formaldehyde Vapor Concentration in Electronic Cigarettes

Formaldehyde was measured in glass container. The measurement of electronic cigarettes brand was done to identify the formaldehyde concentration vapor in each brand of electronic cigarettes. The duration of formaldehyde measurement was 60 minutes within the same smoking period in each measurement. The ingredients of vapor were glycerin (VG), propylene glycol (PG), and nicotine in different amounts. First vapor (local vapor) contained 90% glycerin and 10% propylene glycol. Vapor 2 (USA) contained 60% glycerin and 40% propylene glycol. Vapor 3 (Malay) contained 60% glycerin and 40% propylene glycol. Vapor 4 (local vapor) contained 60% glycerin and 40% propylene glycol. Vapor 5 (Malay) contained 30% glycerin and 70% propylene glycol. Vapor 6 contained 70% glycerin and 30% propylene glycol in electronic cigarettes.

Electronic cigarette generates carbonyl compound in the electronic cigarette smoke mist from the oxidation of liquids such as glycerol and glycols. These liquids are oxidized to formaldehyde, acetaldehyde, acrolein, glyoxal, and methylglyoxal [12]. Thermodegradation of glycerol can lead to various carbonyls, such as acrolein and formaldehyde [13, 14]. Formaldehyde, acetaldehyde, and propionaldehyde were found at room temperature. It showed that at temperature of 150°C the level of formaldehyde and acetaldehyde was higher 20-fold than ambient temperature [15].

Formaldehyde concentration in control was 0.0009 ppm. Six vapors contained formaldehyde in variation concentration with the highest in vapor 1 and vapor 6 (Table 6). Vapor 1 contained 90% glycerin and vapor 6 contained 70% glycerin. Formaldehyde concentration of vapor 5 was 0.0345 which contained propylene glycol 70%. Several studies showed that voltage of electronic cigarette had contributed to difference of formaldehyde concentration. At low voltage (3.3 V) formaldehyde was not detected while it was detected at high voltage (5.0 V) [10]. Propylene glycol was approved by FDA for being applied in some products, but it is not approved for inhalation. When propylene glycol is heated this may change the chemical composition and it may produce propylene oxide in a little amount which is known as carcinogen [11]. Several studies on electronic cigarettes showed an increase formaldehyde concentration [6, 9, 12]. Recent study compared metal concentration in dispenser, tank, and aerosol of electronic cigarettes. It identified and measured aerosol metal concentration due to its toxicity in human health. There were 5 metals measured such as Ni, Cr, Mn, Pb, and As. Ni (57.0%) and Mn (14.0%) concentration in electronic cigarettes exceeded the daily chronic minimum risk level of the Agency for Toxic Substances Disease Registry (median 4.44 x 10^-4 ^mg/m^3^ and 1.97 x 10^-5 ^mg/m^3^, respectively), while Pb (48.0%) concentration in electronic cigarettes exceeded the US EPA National Ambient Air Quality (median 1.06 x 10^-4 ^mg/m^3^). Concentration in aerosol was 25 times higher than in refilling dispenser (Pb and Zn) and was 6 times higher than in the dispenser (Cr, Ni, and Sn). Mn concentration in aerosol was 1.93 times higher than in dispenser. Al, Cd, and Sb concentration was between 1.60 and 3.58 times higher than the dispenser [16]. Electronic cigarettes produced formaldehyde that affected environment especially in the air, while cigarette butts were found to contain Hg and Pb in Persian Gulf Beach. The total of cigarette butts, and Hg and Pb associations of butts, were found to vary between 2 and 38 items per square meter and 2.5 and 86.32 ng/g cigarette butts, as well as 650 and 8630 ng/g cigarette butts, respectively. The abundance of cigarette butts in marine environment is a source of Hg and Pb contamination in coastline area [17].

### 3.3. Health Complaint of Electronic Cigarettes Smoker

The complaints of electronic cigarettes smokers were irritation in nose, eye, and throat. Nose irritation included itchy nose, uncomfortable smell, and sneezing. Eye irritation included watery eye, sore eye, and reddish eye. Respiration complaints included sore throat, dry throat, cough, and asphyxia. The results are shown in Table 7.

Table 8 showed that cotinine urine was mostly positive (88.0%). A study in 28 samples showed cotinine urine among electronic cigarettes smokers was 1,880 (mean) compared to cotinine urine among cigarette smokers in 165 samples who were interested in quitting smoking and were assigned low nicotine cigarettes was 3,930 (mean) [18]. The result showed that mostly prevalent complaint was upper airway irritation (dry throat). It was due to vapor smoked from electronic cigarettes. Nose irritation mostly was sneezing while eye irritation was rare. Propylene glycol is known to cause upper airway irritation [19]. A study reported that using electronic cigarette had immediate adverse physiologic effects after short-term use which were similar to some effects of tobacco smoking [20]. The health effect of electronic cigarette is influenced by the safety and quality of liquid [21]. It is due to the fact that the solvent of liquid may remain on available surface [22].

Health effect of nicotine and other aerosols that come from electronic cigarettes is not well understood. A research showed that electronic cigarettes aerosol is having a risk for person who has a compromised health condition and limited ventilation. Flavoring availability is for food product not for liquid electronic cigarettes flavoring. There is limited data about the health effect of inhaled flavorings and the synergistic effect from electronic cigarettes contents and environmental contaminants [23]. In Indonesia, there is no regulation of electronic cigarettes yet. While, in 2016, in Great Britain the regulation of electronic cigarettes as a nonprescription medicine started, other countries such as Brazil, Singapore, and Norway have banned the use of electronic cigarettes [5]. Respondent's opinion on policy of electronic cigarette in Indonesia was mostly “do not agree” if there is a policy to restrict electronic cigarette usage. The regulation of electronic cigarettes varies in 33 countries. WHO conducted a survey in 2011 about regulation and availability of electronic cigarettes within their country, 13 reported no availability, 16 reported they were available (nine unregulated, seven with some type of regulation), and four were unsure [24]. An analysis was undertaken of key elements in electronic cigarettes position statements from selected national/international health agencies such as general public health risk assessment and explicit statement that consumers should avoid electronic cigarettes. General public health risk assessment about electronic cigarettes was stated from Non-Smokers' Rights Association (Canada), Campaign for Tobacco Free Kids (USA), Canada Lung Association, and Cancer Society (New Zealand). The explicit statement that consumers should avoid electronic cigarettes was stated from WHO, Scottish Dir Public Health [5]. There are limited data on the effects of recurrent long-term exposures to aerosolized nicotine, flavoring, and propylene glycol. The effects of an aerosol delivery system on the quantity of nicotine consumed by users are unknown. Electronic cigarettes have the potential for significant impact on public health. At this time, data are not sufficient to confirm a long-term benefit for users or a public health benefit for the population at large [25].

The limitation of this research was lack of electronic cigarettes brand distributed in Indonesia, from Indonesia and from other countries as well. Because of it, the determination of electronic cigarettes as samples in this research was not various. Exposure of electronic cigarette related to health complaints was not identified toxicologically. The other chemical measurements in electronic cigarettes were not identified.

## 4. Conclusion

In conclusion, mostly the first time users smoked electronic cigarette was at 2015, smoking frequency of electronic cigarettes was mostly twice a day and three times a day. Health complaints were mostly upper airway irritation with acute effect and mostly cotinine urine was positive. The recommendations are to educate users about hazard of electronic cigarettes due to the regulation of electronic cigarettes not being set yet and to conduct further research to identify chemical agents in electronic cigarettes.

## Figures and Tables

**Table 1:** Characteristics of cigarette smoker in personal vapor community in Surabaya city in 2016.

**Age (years)**	**Percentage (**%**)**

18	12.0
19	20.0
20	16.0

**Age (years)**	**Percentage (**%**)**

21	8.0
22	8.0
23	8.0
24	12.0
25	12.0

Source: primary data.

**Table 2:** Education and occupation of electronic cigarettes smoker in personal vapor community in Surabaya city in 2016.

**Education**	**Percentage (**%**)**

Bachelor	12.0
High School	80.0
Master	8.0

**Occupation**	**Percentage (**%**)**

Athlete	4.0
College student	44.0
Entrepreneur	8.0
Labor	4.0
Private worker	36.0
Unemployment	4.0

Source: primary data.

**Table 3:** Onset of using electronic cigarettes among personal vapor community in Surabaya city in 2016.

**Year**	**Percentage (**%**)**
2008	4.0
2012	4.0
2013	4.0
2014	28.0
2015	36.0
2016	24.0

Source: primary data.

**Table 4:** Frequency of daily smoking of electronic cigarettes in personal vapor community in Surabaya city in 2016.

**Frequency (time)**	**Percentage (**%**)**
1	8.0
2	12.0
3	12.0
4	8.0
5	4.0
6	8.0
11	4.0
12	4.0
20	4.0
Unclassified	36.0

Source: primary data.

**Table 5:** Reasoning of smoking electronic cigarettes in personal vapor community in Surabaya city in 2016.

**Reasoning**	**Percentage (**%**)**
Harmless	4.0
Liking	4.0
To quit smoking conventional cigarettes	36.0
To reduce smoking conventional cigarettes	12.0
To taste the flavor	20.0
To try	24.0

Source: primary data.

**Table 6:** Formaldehyde vapor concentration in electronic cigarettes.

**Measurement**	**Formaldehyde Concentration (ppm)**

Control	0.0009
1	0.1490
2	0.0658
3	0.0767

**Measurement**	**Formaldehyde Concentration (ppm)**

4	0.0709
5	0.0345
6	0.1419

Source: primary data.

**Table 7:** Health complaint of electronic cigarettes smoker in personal vapor community in Surabaya city in 2016.

**Health Complaint **	

**Eye Irritation**	**Yes (**%**)**

Reddish eye	0 (0.0)
Sore eye	1 (5.0)
Watery eye	1 (5.0)

**Nose Irritation**	**Yes (**%**)**

Itchy nose	3 (15.0)
Sneezing	4 (20.0)
Uncomforted smell	1 (5.0)

**Upper airway irritation**	**Yes (**%**)**

Asphyxia	1 (5.0)
Cough	5 (25.0)
Dried throat	13 (65.0)
Sore throat	2 (10.0)

Source: primary data.

**Table 8:** Cotinine urine of electronic cigarettes smoker in personal vapor community in Surabaya city in 2016.

**Cotinine Urine**	**Percentage (**%**)**
Negative	12.0
Positive	88.0

Source: primary data.

## Data Availability

Data can be accessed by contacting the corresponding author. No data were used to support this study.
